# Association of environmental factors and high HFMD occurrence in northern Thailand

**DOI:** 10.1186/s12889-020-09905-w

**Published:** 2020-11-30

**Authors:** Pussadee Laor, Tawatchai Apidechkul, Siriyaporn Khunthason, Vivat Keawdounglek, Suntorn Sudsandee, Krailak Fakkaew, Weerayuth Siriratruengsuk

**Affiliations:** 1 School of Health Science, Mae Fah Luang University, Chiang Rai, Thailand; 2 Center of Excellence for the Hill tribe Health Research, Mae Fah Luang University, Muang Chiang Rai, Thailand

**Keywords:** Children, Day care center, Environment and sanitation, HFMD, Hygiene

## Abstract

**Background:**

The major population vulnerable to hand, foot and mouth disease (HFMD) is children aged less than 5 years, particularly those who are cared for at day care centers (DCCs). This study aimed to assess the associations of environmental and sanitation factors with high HFMD occurrence rates in DCCs of northern Thailand.

**Methods:**

A case-control study was used to gather information from caregivers and local government administrative officers. DCCs in areas with high and low HFMD occurrence rates were the settings for this study. A validated questionnaire was used to collect environmental and sanitation information from the DCCs. In-depth interviews were used to collect information from selected participants who were working at DCCs and from local government administrative officers on the HFMD capacity and prevention and control strategies in DCCs. Logistic regression analysis was used to determine the associations between many environmental factors and HFMD at the α = 0.05 significance level while the content analysis was used to extract information from the interviews.

**Results:**

Two variables were found to be associated with a high rate of HFMD occurrence: the number of sinks available in restrooms and the DCC size. Children attending DCCs that did not meet the standard in terms of the number of sinks in restrooms had a greater chance of contracting HFMD than children who were attending DCCs that met the standard (AOR = 4.21; 95% CI = 1.13–15.04). Children who were attending a large-sized DCC had a greater chance of contracting HFMD than those attending a small-sized DCC (AOR = 3.28; 95% CI = 1.21–5.18). The yearly budget allocation and the strategies for HFMD control and prevention, including collaborations among stakeholders for HFMD control and prevention in DCCs, were associated with the effectiveness of HFMD control and prevention.

**Conclusions:**

The number of sinks in restrooms and DCC size are major concerns for HFMD outbreaks. Sufficient budget allocation and good collaboration contribute to effective strategies for preventing and controlling HFMD in DCCs.

## Background

Hand, foot and mouth disease (HFMD) is a common communicable disease among children. In 2018, more than 1.6 million cases were reported in the western Pacific regions (China, Japan, Korea, Singapore and Vietnam) [1]. HFMD has become a major public health threat, especially among people living in tropical zones [2]. Several health resources have been used for treatment, care, prevention and control. People living in crowded places in rural areas with poor sanitation and hygiene conditions, including limited access to a sufficient water supply, are at a high risk for HFMD infection [3, 4]. Many serious complications, particularly in the nervous system, have been reported for HFMD patients [5]. All HFMD patients including those who present with mild and moderate symptoms, they must be properly observed and treated by a medical doctor to prevent death [6]. HFMD is caused by viruses, such as enterovirus A71 [7, 8] and coxsackievirus [9–11], then a specific treatment is therefore not available.

Many studies have shown that HFMD is related to environmental and sanitation factors, such as high environmental temperatures [11], high rainfall volumes [12, 13], high wind speeds [12], antiseptic availability and personal hygiene conditions [14], including the density of people living in an area or in a family and poor sanitation [15]. In 2018, the Ministry of Public Health, Thailand reported that more than 70,000 cases of HFMD and 3 deaths [16]. Most people affected by the disease were children aged less than 5 years [16–18]. The northern region; Chiang Rai, Chiang Mai, and Pha Yao Provinces, was classified as having the highest incidences of HFMD at 279.72, 279.12, and 321.24 per 100,000 population, respectively [10, 17, 19]. Due to specific geographical characteristics of the Chiang Rai Province, including long border lines with China, Myanmar, and Laos [20, 21] and heavy population migration, people of this province are at a greater risk of HFMD infection than those living in other provinces or regions of Thailand [20]. A large proportion of people living in the Chiang Rai Province, especially the hill tribe people, have low economic profiles and low educational attainment [10]. Hill tribe people have their own traditional lifestyle, particularly in regards to their planting of crops that are not traditionally thought to be economical [22]. Almost 30.0% of all people living in the Chiang Rai Province of Thailand are of hill tribes [23]. Hill tribe people have been indicated to be particular vulnerable to HFMD infection [20], and most live in multigeneration families [23]. Driven by their economic status, most hill tribe people leave their children at day care centers (DCCs) during the day while they work on the farms.

DCCs are organized by local governments, where all children attending are cared for in small spaces [24, 25]. Many local governments have tried to support both financial and physical infrastructure developments to operate DCCs. However, infrastructures and sanitation factors, such as the number of restrooms and other measures to control and prevent tropical diseases such as HFMD in children, are not adequately supported. As a result, many HFMD cases are reported to originate from DCCs and to lead to economic and life losses [17, 26].

## Methods

**Fig. 1:**
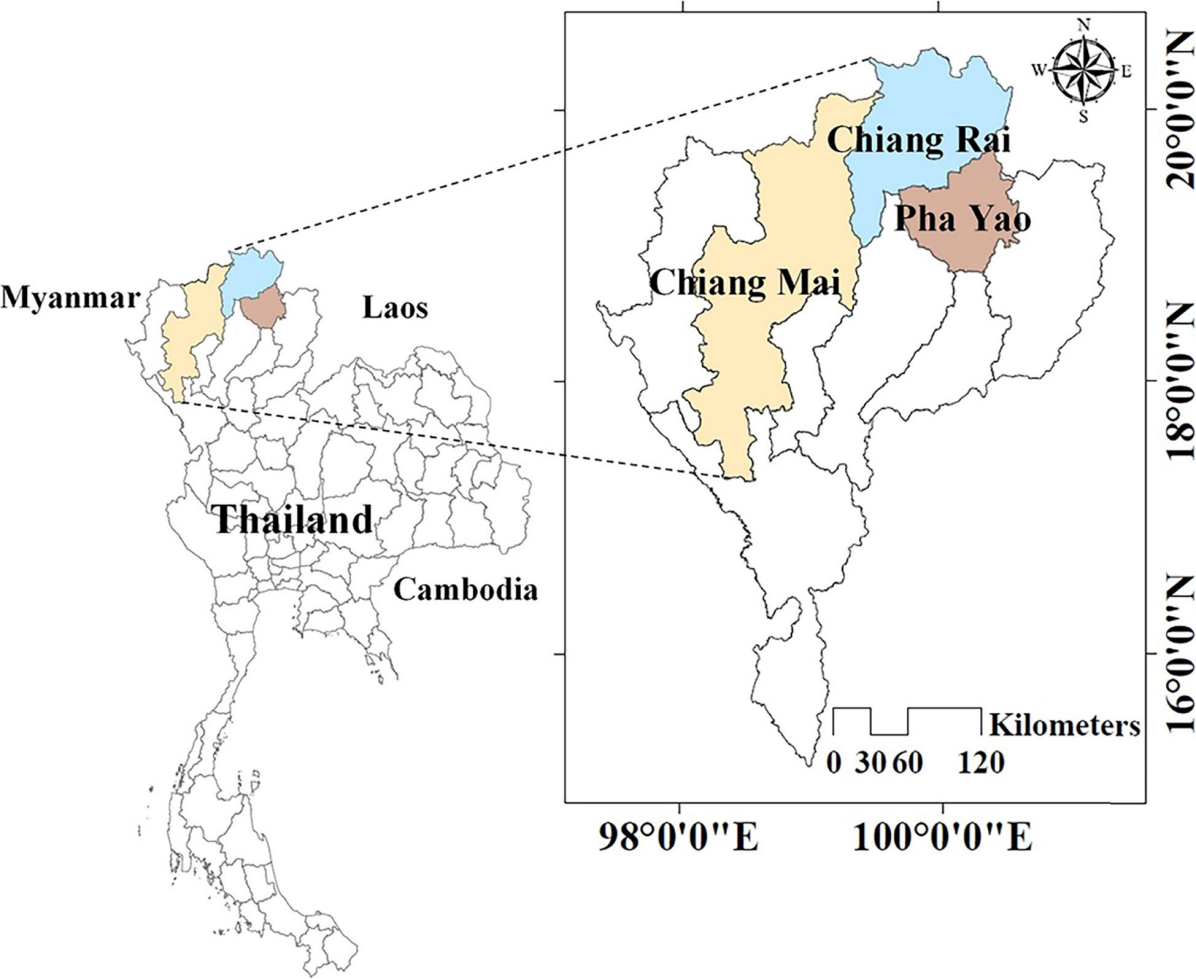
Study settings; Chiang Rai, Chiang Mai, and Pha Yao Provinces, Northern Thailand

A case-control study was conducted in three districts of provinces in the northern part of Thailand (Chiang Mai, Chiang Rai and Pha Yao), with high and low numbers of HFMD cases (Fig. 1). The environmental and sanitation information regarding the DCCs was collected according to the questionnaire developed. The criterion for classifying the areas of high and low HFMD case numbers was the median number of HFMD cases reported in the three previous years, which was 146 per 100,000 individuals [10].

**Fig. 2:**
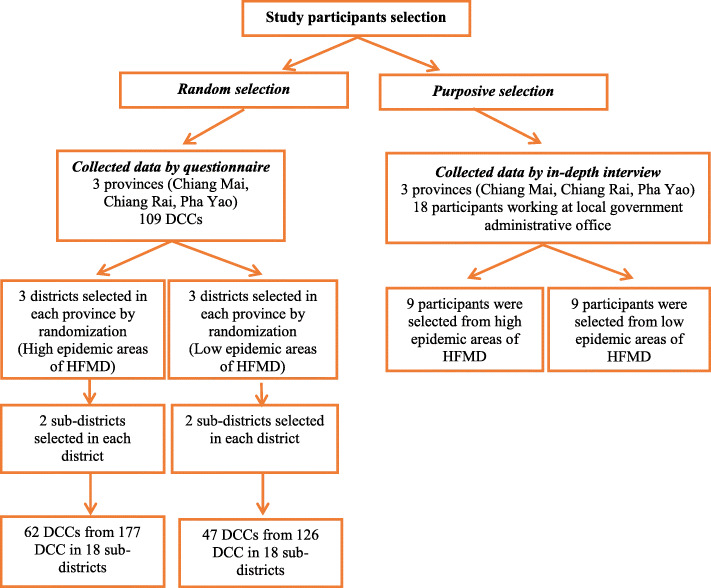
Flow of selection the study participants

A computer generated number method was used to select 62 DCCs from the 177 centers with high HFMD case numbers (case area) and 47 DCCs from the 126 centers with low HFMD case numbers (control area). Moreover, information on the capacity and management of HFMD disease prevention and control strategies during an outbreak at DCCs was also collected by in-depth interviews of local government officers; 9 participants from 3 areas with high HFMD case numbers and 9 participants from 3 areas with low HFMD case numbers were interviewed (Fig. 2).

Questionnaires were developed and used for data collection in the DCC units. This study was not intended to assess individual characteristics, which have commonly and largely been reported globally, but rather was developed from a literature review and discussion with experts in the field and consisted of four parts: environmental and sanitation factors, cleaning procedures in the DCCs, personal hygiene, and open-ended questions to obtain information on capacity and management strategies for HFMD prevention and control. In part one, 10 questions were used to collect information regarding environmental and sanitation factors, such as the indoor lighting level (the percentage of lights within each DCC that met the indoor lighting standard), air ventilation, and first aid locations. In part two, 7 questions were used to collect data related to cleaning procedures (cleaning glasses, utensils, beds, etc.). In part three, 4 questions were used to collect data on personal hygiene (hand washing before meals and after using the restroom, etc.). In the last part, the following 3 open-ended questions were used to collect data on the capacity and management strategies before, during, and after an HFMD epidemic: 1) Do you have a specific team to respond to HFMD prevention and control? 2) How did you prevent and control HFMD outbreaks before, during and after this epidemic? and 3) Did you have any previous limitations for HFMD prevention and control? (Additional file 1, Questionnaire).

All questions were examined for validity and reliability by the item-objective congruence (IOC) method [27] and verified by three external experts in the field: an epidemiologist, a medical doctor, and an environmentalist. Afterward, a pilot test was conducted at 4 DCCs with similar characteristic samples in the Mae Chan district of the Chiang Rai Province to test the feasibility. Finally, all the questions were reviewed by the research team before the project began.

After selecting the DCCs, the subdistrict local government officers were contacted to acquire their approval to access and contact the directors of the selected DCCs. Once permission was given by the local administration officers in the district, the directors of the selected DCCs were contacted, and the appointments were made. All essential information regarding the study was explained to the DCC directors, and written informed consent was obtained from all participants before data collection began. The DCCs were observed and assessed according to the questionnaire, and the directors and relevant staff were thereafter invited to provide additional information via the open-ended questions. The entire data collection process required one hour at each DCC.

Two people entered the same interview data into separate Excel sheets and checked for errors before starting the analysis to ensure that the data were of good quality before being analyzed. The data were analyzed using SPSS (version 11.5; 2006 SPSS, Chicago, Illinois) and secured with a specific password accessible by only the researchers. Chi-square and logistic regression analyses were used to determine the associations between environmental factors and HFMD at DCCs at the α = 0.05 significance level. When performing the logistic regression analysis, the “Enter” mode was used to select the variables in the model, which allowed the researcher to consider the fit of the model before and after removing a variable from the model. Moreover, the Hosmer-Lemeshow Chi-square, Cox-Snell R^2^ and Nagelkerke R^2^ parameters were used to assess the model and quality of the outcome predictions at each step of the analysis. Data from the open-ended questions were analyzed for content to extract key and important information, which was performed by all researchers.

For the content analysis step, all interviews were taped, and the transcripts were typed and checked for errors. Afterward, the typed documents were sent to all of the research teams to make them familiar with the content and to allow information to be preliminarily extracted from the documents. Additionally, the data were extracted and analyzed according to themes using the NVivo program (NVivo, qualitative data analysis software; QSR International Pty Ltd., version 11, 2015), and the findings were discussed before conclusions were drawn.

## Results

A total of 109 DCCs were recruited into the study, 62 from areas with high HFMD case numbers and 47 from areas with low HFMD case numbers. Among the DCCs from areas with high HFMD case numbers (case group), 56.5% were small in size (≤ 50 children), 56.5% had at least one hill tribe child, and 54.8% were located in urban fringe areas. Regarding the ratio of caregivers to children, 45.2% had ratios of 1 to 11–20 children, followed by 1 to 10 children (43.5%). Most of the DCCs (76.6%) from areas with low HFMD case numbers (control group) were small in size, while 5.3% had at least one hill tribe child, and 61.7% were located in urban areas. The largest group had a 1:10 ratio of caregivers to children (48.9%). In the chi-squared test, only the DCC size was associated with high HFMD case numbers (Table 1).

**Table 1:** Comparisons of general DCC characteristics between the areas with high and low HFMD case numbers

Characteristics	Areas with high-HFMD incidence	Areas with low HFMD incidence	***χ***^***2***^	***p***-value
	***n*** (%)	***n*** (%)		
**Total**	***62 (100.0)***	***47 (100.0)***		
***DCC sizes based on number of children***				
Small (≤ 50 children)	35 (56.5)	36 (76.6)	7.266	0.026^a^
Medium (51–80 children)	17 (27.4)	10 (21.3)		
Large (> 80 children)	10 (16.1)	1 (2.1)		
***At least one hill tribe child***				
Yes	35 (56.5)	26 (55.3)	0.014	0.906
No	27 (43.5)	21 (44.7)		
***Location***				
Urban	34 (54.8)	29 (61.7)	0.343	0.898
Rural	28 (45.2)	18 (38.3)		
***Ratio of a caregivers to children*** *(persons)*				
1: ≤10	27 (43.5)	23 (48.9)	1.787	0.409
1: 11–20	28 (45.2)	22 (46.8)		
1: ≥21	7 (11.3)	2 (4.3)		

^a^Significance level at α = 0.05

In the univariate analysis, four variables were found to be associated with areas of high HFMD incidence in DCCs: indoor light, first aid room, number of sinks in restrooms, and large size. DCCs with a high rate of having met the indoor light standard were less likely to have high HFMD numbers than those with a low rate of having met the indoor light standard (OR = 0.27; 95% CI = 0.12–0.60). DCCs that had first aid available were less likely to be in the high HFMD case report group than those with no first aid rooms available (OR = 0.39; 95% CI = 0.17–0.89), and DCCs that did not have enough sinks in restrooms for children were more likely to be in the high HFMD case report group than DCCs with enough sinks in restrooms for children (OR = 3.72; 95% CI = 1.61–8.62) (Table 2).

**Table 2:** Univariate and multivariate analyses of factors associated with areas with high HFMD case numbers

Factors	High HFMD incidence ***n*** (%) ***compliance***	Low HFMD incidence ***n*** (%) ***compliance***	Univariate analysis			Multivariate analysis		
			OR	95% CI	***p***-value	AOR	95% CI	***p***-value
**Total**	**62 (100.0)**	**47 (100.0)**	**N/A**	**N/A**	**N/A**	**N/A**	**N/A**	**N/A**
*(1) Indoor environment*								
Indoor light	42 (67.7)	17 (36.2)	0.27	0.12–0.60	0.001^a^			
Area of windows and doors	56 (90.3)	42 (89.4)	0.90	0.26–3.15	0.869			
Ventilation	56 (90.3)	43 (91.5)	1.15	0.31–4.34	0.835			
Area for activities	54 (87.1)	37 (78.7)	0.55	0.20–1.52	0.248			
First aid room	29 (46.8)	12 (25.5)	0.39	0.17–0.89	0.025^a^			
Sufficient toys for children	57 (91.9)	43 (91.5)	0.94	0.24 t-3.72	0.933			
Personal amenities	52 (83.9)	42 (89.4)	1.62	0.51–5.09	0.413			
Number of toilets	39 (62.9)	32 (68.1)	1.26	0.56–2.80	0.574			
Number of sinks in the restroom	29 (46.8)	36 (76.6)	3.72	1.61–8.62	0.002^a^	4.12	1.13–15.04	0.032^a^
Hand soap in the restroom	44 (70.1)	37 (78.7)	1.51	0.62–3.68	0.360			
*(2) Frequency of cleaning utensils, toys and some spaces or areas inside the building with a cleaning solution*								
Frequency of cleaning glass	53 (85.5)	37 (78.7)	0.63	0.23–1.70	0.359			
Frequency of cleaning handkerchiefs and napkins	26 (41.9)	18 (38.3)	0.86	0.40–1.87	0.702			
Frequency of cleaning bed sheets, pillowcases and blankets	62 (100.0)	45 (95.7)	0.15	0.01–3.11	0.217			
Solution used for cleaning toys	59 (95.2)	42 (89.4)	0.43	0.10–1.87	0.262			
Frequency of cleaning mouth-use toys	2 (3.2)	3 (6.4)	2.05	0.33–12.76	0.444			
Frequency of cleaning inside building areas or spaces	59 (95.2)	45 (95.7)	1.14	0.18–7.14	0.885			
Proper cleaner types	62 (100.0)	44 (93.6)	0.10	0.01–2.02	0.134			
*(3) Personal hygiene*								
Hand washing before meals	54 (87.1)	36 (76.6)	0.48	0.18–1.32	0.157			
Hand washing after using the toilet	10 (16.1)	7 (14.9)	1.22	0.57–2.61	0.612			
Hand washing after playing with toys	14 (22.6)	12 (25.5)	1.18	0.48–2.85	0.720			
Separate drinking glass	26 (41.9)	22 (46.8)	1.22	0.57–2.61	0.612			
*(4) DCC size*								
Large DCC size (> 80 children)	10 (16.1)	1 (2.1)	10.29	1.25–84.64	0.032^a^	3.28	1.21–5.18	0.041^a^

^a^Significance level at α = 0.05

In the multivariate model, two variables were found to be associated with high HFMD case numbers in DCCs: the number of sinks in restrooms and the DCC size. Children who attended DCCs that did not meet the standards for the number of sinks in restrooms had a greater chance of contracting HFMD than those who attended DCCs that met the standard for the number of sinks in restrooms (AOR = 4.21; 95% CI = 1.13–15.04). Children who attended large-sized DCCs had a greater chance of contracting HFMD than those who attended small-sized DCCs (AOR = 3.28; 95% CI = 1.21–5.18) (Table 2).

### Comparisons of the capacities and responses to HFMD epidemics between the areas with high and low HFMD case numbers

Several differences were observed in the context of HFMD prevention and control between the areas with high and low HFMD case numbers. First, in the areas with high HFMD case numbers, the DCCs did not have a specific team or person appointed to respond to HFMD outbreaks and did not regularly having meetings regarding prevention and control strategies, particularly before the financial beginning of the calendar year (October–February). In the areas with low HFMD case numbers, specific teams responsible for responding to HFMD outbreaks and for prevention and control strategies were documented. Moreover, in the areas with low HFMD case numbers, most of the DCCs had a proper plan and chemicals to use in response to HFMD epidemics, unlike those in the areas with high HFMD case numbers. However, a few DCCs in areas with high HFMD case numbers did plan and properly prepare for HFMD prevention and control.

During the HFMD epidemic, most of the DCCs in areas with high HFMD case numbers used improper disinfectants or concentrations to respond to the epidemic. In the areas with low HFMD case numbers, public health professionals from the subdistrict health-promoting hospitals frequently joined in on the activities to prevent and control HFMD; they used proper strategies to prevent and control the disease, including correctly using disinfectants, and these centers could therefore completely control the epidemic within a short period. The staff at DCCs in areas with low HFMD case numbers had better HFMD prevention and control strategies than those in areas with high HFMD case numbers.

Regarding the limitations for HFMD prevention and control, there were a few problems in the areas with low HFMD case numbers, such as large-sized DCCs, and some parents had little knowledge and poor skills regarding good hygienic care for their children. In the areas with high HFMD case numbers, many points were found to be weaknesses for HFMD prevention and control. First, the strategy and collaboration among DCC staff, local government staff, and public health workers from subdistrict health-promoting hospitals was not optimal. Second, no annual budget was allocated for this purpose at DCCs in areas with high HFMD case numbers, unlike those in areas with low HFMD case numbers, which had proper budgets allocated for this purpose. Third, no HFMD prevention and control procedures were in place for DCCs in the areas with high HFMD case numbers. Last, the perception and attitudes regarding HFMD control among the executive teams in areas with high HFMD case numbers were worse than those at DCCs in areas with low case numbers.

## Discussion

A large size, based on the number of children, and having few sinks in a restroom were significantly associated with high HFMD case numbers in DCCs in northern Thailand, and the executive members of DCCs in these areas had poor knowledge and attitudes regarding disease control and prevention. Moreover, the yearly budget allocation and strategies for HFMD control and prevention, including collaborations among stakeholders, were obviously associated with the effectiveness of HFMD control and prevention in DCCs in northern Thailand.

In our study, the DCC size, based on the number of children, was a significant factor associated with HFMD in northern Thailand, and this result coincides with a study conducted in China reporting that children living in high-density areas had a greater chance of HFMD infection than those living in low-density areas [28]. Two more studies in China clearly demonstrated the association between population density and the occurrence of HFMD [29, 30]. However, a study in Bangkok, Thailand reported that the most important factor underlying HFMD outbreaks was the number of close contacts with infected people [31], while density was not found to be associated with HFMD. This difference might be attributed to that fact that although the areas in our study are close to China and have a similar climate, China still differs substantially from Bangkok, Thailand. Additionally, a study in China reported that most of the serious HFMD serotypes were found in crowded areas, particularly in DCCs [32], and a multipeak HFMD epidemic was found in high-density areas [33].

Another factor that was found to be associated with high HFMD case numbers in DCCs in northern Thailand was the sanitary limitations in restrooms. The DCCs with few or limited numbers of sinks in restrooms exhibited high HFMD case numbers. This result might be due to the face that DCCs with no sinks or limited sinks available tend to limit handwashing among the children. As a result, HFMD outbreaks could increase due to irregular hand washing among children in the DCCs. Several studies [34–36] have reported that hand washing is a major behavior performed by children that can be used to predict HFMD epidemics in DCCs, with children who are less engaged in hand washing being associated with more HFMD cases.

This study does have a few limitations. First, many DCC executives from the areas with high HFMD case numbers were more likely to poorly cooperate with providing essential information regarding HFMD prevention and control. Researchers asked other relevant people to obtain the information required. Some of the parents could not provide essential information regarding the study protocol, particularly those who were from hill tribes (more than half of the children in the areas of both high and low HFMD case numbers were from hill tribes); thus, we used proxy information that was obtained from caregivers and their neighborhoods to complete the information before the analysis. As mentioned in the methods section, this study focused on assessing the environmental and sanitation factors regarding DCCs instead of on individual characteristics of both children and parents and was thus focused on the larger factors contributing to the problem. Additional integrative investigations of personal variables and environmental factors related to the prediction of HFMD occurrence would be optimal for the effective development of public health intervention strategies for HFMD prevention and control.

## Conclusion

Children attending DCCs in northern Thailand face the problem of HFMD infection, particularly those attending large DCCs with limited sanitation strategies. The management skills of executives, including their personal attitudes, regarding the care of children in DCCs impact the possibility of HFMD outbreaks. DCC infrastructures need to be assessed and improved to meet the quality standards, and good infectious disease management guidelines and effective strategies for preventing and controlling particularly serious communicable diseases are required to reduce the number of cases and lives lost.

## Supplementary Information

**Additional file 1.** Questionnaire

## Data Availability

Additional data are available from the corresponding author upon request.

## Abbreviations

CI: Confidence interval
DCC: Day care center
HFMD: Hand foot mouth disease
IOC: Item-objective congruence
OR: Odds ratio
AOR: Adjusted odds ratio
WHO: World Health Organization
