# Supplementary material for: Association of environmental factors and high HFMD occurrence in northern Thailand
Source: BMC Public Health. 2020 Nov 30;20:1829. doi: 10.1186/s12889-020-09905-w (PMC7706220; doi:10.1186/s12889-020-09905-w)
Supplement: Supplementary file 1 — Additional file 1. Questionnaire [file 12889_2020_9905_MOESM1_ESM.pdf]

Environmental and Hygiene Management in Day Care Center (DCC)

Number of caregivers…………………… People; Number children…………………..people

Number of HFMD ………………………cases one year prior.

Part I Internal and external environment

1. The indoor light in the building

( ) 1. There is light throughout the room

( ) 2. There is light in some corners of the room.

( ) 3. Insufficient lighting, dark appearance

( ) 4. Others………………………………….

2. Areas of windows and doors in various rooms of the DCC

( ) 1. A door window of less than 20% of the room area.

( ) 2. A door window more than 20% of the room area.

( ) 3. Others ………………………………….

3. Characteristics ventilation. Which of the following characteristics was found (can select more than one answer)

( ) 1. Has a musty smell

( ) 2. No wind blowing through at all. Poorly ventilated

( ) 3. Good ventilation, not found the characteristics according to items 1 and 2

4. Space for learning activities of children

( ) 1. Less than 1 square meter per person

( ) 2. Less than 2 square meters per person

( ) 3. About 2 square meters per person.

( ) 4. Others ……………………………………………….

5. First aid room in DCC.

( ) 1. There is no first aid room.

( ) 2. There is a first aid room for caring sick children, but not separated from the not sick children.

( ) 3. There is a first aid room for caring sick children, separated from those who are not sick.

( ) 4. Others ………………………………………….

6. The number of toys in children's learning activities.

( ) 1. 1 piece per 1 child

( ) 2. 1 piece per 2 children

( ) 3. 1 piece per more than 2 children

( ) 4. Others …………………………………………………………

7. Personal amenities for children, such as combs, toothbrushes, drinking glasses, towels.

( ) 1. All children have all personal items and has the name or identification symbol embroidered.

( ) 2. All children have all personal items but no name or identification.

( ) 3. Children do not have personal items and share personal amenities with others in the room

( ) 4. Others ……………………………………………………………

8. Toilets room per number children

( ) 1. More than 12 children per 1 toilet room

( ) 2. Not more than 12 children per 1 toilet room

9. Sink in toilet room, which of the following equipment or features?

( ) None

( ) If have. Number of sinks……………………………….

10. Hand soap for children.

( ) 1. None

( ) 2. If have, stated that it is for children / brands ………………………………….

( ) 3. If have, but it is not indicated for use by children / brands ……………………………

( ) 4. Others …………………………………………………

Part II Cleaning of materials, appliances and toys for children / building

Item 1: Glass, handkerchief, personal use

1. The cleaning frequency of drinking water glass with a cleaning solution such as dishwashing liquid

   ( ) 1. Everyday ( ) 2. Every 2 - 3 days

   ( ) 3. Every week ( ) 4. Others …………………………………….

2. The cleaning frequency of handkerchief and napkin with detergent and dry with sunlight

   ( ) 1. Everyday ( ) 2. Every 2 - 3 days

   ( ) 3. Every week ( ) 4. Others …………………………………….

Item 2: Cleaning solution and cleaning frequency for utensils, toys and indoor building

3. The cleaning frequency of bed sheets, pillowcases and blankets.

( ) 1. Every week ( ) 2. Every two weeks ( ) 3. Every month ( ) 4. Others ……………

Item 3: Children toys

4. The cleaning products for children toys.

( ) 1. It is a cleaning solution type for children

( ) 2. Use detergent and soap to clean

( ) 3. Use dishwashing liquid to clean

( ) 4. Others …………………………………………………………

5. The frequency of cleaning of mouth-use-toys

( ) 1. Everyday ( ) 2. Every week

( ) 3. Every month ( ) 4. Others ………………………………

Issue 5 : Indoor cleaning

6. The frequency of cleaning the floors and walls of bedrooms, play rooms, classrooms are cleaned with cleaning agents or antiseptic.

( ) 1. Everyday ( ) 2. Every 2 - 3 days

( ) 3. Every week ( ) 4. Others …………………………………….

7. Cleaners solution used to clean the building.

( ) 1. Water ( ) 2. Cleaners solution or disinfectant

( ) 3. Others ………………………………………………………..

Part III Personal hygiene care

Statement: Please check / in the box next to the action.

( ) 1. Caregiver provide children to wash their hands with soap or hand sanitizer every time before - after eating.

( ) 2. Caregiver provide children wash their hands with soap or gel, wash your hands after the toilet.

( ) 3. Caregiver provide children to wash their hands with soap or gel to wash hands every time after play toys.

( ) 4. All children have their own drinking glasses and do not share glasses with other children.

Part IV Opened questions (This part is used for in-depth interview in selected participants)

1) Do you have a specific team to respond to the HFMD prevention and control?

2) How did you to prevent and control before, during and after HFMD epidemic?

3) Did you have any previous limitations for HFMD prevention and control?
